# EU Regulation of Nanobiocides: Challenges in Implementing the Biocidal Product Regulation (BPR)

**DOI:** 10.3390/nano6020033

**Published:** 2016-02-16

**Authors:** Anna Brinch, Steffen Foss Hansen, Nanna B. Hartmann, Anders Baun

**Affiliations:** 1 Faculty of Science, University of Copenhagen, Bülowsvej 17, DK-1870 Frederiksberg C, Denmark; annabrinch@gmail.com; 2 Department of Environmental Engineering, Technical University of Denmark, DTU-Building 113, DK-2800 Copenhagen, Denmark; nibh@env.dtu.dk (N.B.H.); abau@env.dtu.dk (A.B.)

**Keywords:** biocides, nanomaterials, European Union (EU) policy, regulation, risk assessment, ecotoxicity

## Abstract

The Biocidal Products Regulation (BPR) contains several provisions for nanomaterials (NMs) and is the first regulation in the European Union to require specific testing and risk assessment for the NM form of a biocidal substance as a part of the information requirements. Ecotoxicological data are one of the pillars of the information requirements in the BPR, but there are currently no standard test guidelines for the ecotoxicity testing of NMs. The overall objective of this work was to investigate the implications of the introduction of nano-specific testing requirements in the BPR and to explore how these might be fulfilled in the case of copper oxide nanoparticles. While there is information and data available in the open literature that could be used to fulfill the BPR information requirements, most of the studies do not take the Organisation for Economic Co-operation and Development’s nanospecific test guidelines into consideration. This makes it difficult for companies as well as regulators to fulfill the BPR information requirements for nanomaterials. In order to enable a nanospecific risk assessment, best practices need to be developed regarding stock suspension preparation and characterization, exposure suspensions preparation, and for conducting ecotoxicological test.

## 1. Introduction

Nanomaterials (NM) are increasingly being incorporated into consumer products such as textiles and paints to provide a biocidal effect. Regulatory bodies in Europe have recognized that there is a need for legislation within the field of nanotechnology, and specific provisions regarding NMs are now being introduced into different legislations in the European Union. The Cosmetic Products Regulation was the first European legislation to include specific provisions regarding labeling of products containing NMs, and similar ingredient labeling provisions were later included in the EU regulation for food information to consumers [1,2,3].

In 2013, the European Biocidal Product Regulation (BPR) came into force. This regulation includes even more comprehensive provisions regarding NMs compared to the Cosmetic Products Regulation, and is the first European regulation to include specific testing requirements of NMs as a part of the information requirements [4]. The BPR covers not only active substances that are used to suppress the growth of organisms, which are harmful to human or environmental health, but also biocidal products and treated articles [5]. Biocidal products are defined as: (1) any substance or mixture consisting of, containing or generating one or more active substances; and (2) any substance or mixture, generated from substances or mixtures, which do not themselves fall under the former category of biocidal products. A treated article shall be considered a biocidal product if it has a primary biocidal function according to the Biocide Pesticide Regulation (BPR) [4].

It is well established that nanomaterials pose unique challenges for ecotoxicological testing using the currently available guideline tests [6,7,8,9,10,11,12] and is not clear exactly how manufacturers of biocidal nanomaterials can fulfill the nanospecific requirements of the BPR due to these methodological challenges.

In this paper, we focus on the EU regulation of nanobiocidal products, summarize the nanospecific requirements of the Biocide Pesticide Regulation (BPR), and analyze how the BPR requirements could be fulfilled for the case of copper oxide (CuO) nanoparticles (NPs) using the open literature. This manuscript includes a discussion of relevant Organisation for Economic Co-operation and Development (OECD) Guidance documents and scientific information regarding aquatic ecotoxicity testing of metal NPs. Finally, we provide a discussion of the deficiencies of existing test guidelines and the challenges facing manufacturers and regulators in implementing the BPR requirements for nanomaterials.

## 2. The BPR and the Introduction of Nanospecific Provisions

Specific requirements regarding NMs were not included in the first draft of the legislative proposal for a biocide regulation from the European Commission [13]. It was not until the first reading in the European Parliament (EP) that specific provisions regarding NMs were proposed. The EP decided to include nano-specific provisions in the BPR due to scientific uncertainty about the safety of NMs [14]. This was based on a report from the Scientific Committee on Emerging and Newly Identified Health Risks (SCENIHR), which had identified health hazards and toxic effects of some NMs. The report furthermore found that high-quality data on exposure of both humans and the environment were lacking and concluded that the methods for exposure estimates and hazard identification for NMs needed to be further developed and standardized. Based on this information, along with the fact that the use of NMs in biocidal product may increase as the technology develops, the EP decided to accept the BPR under the condition that a series of nano-specific provisions were introduced [14]. The Council of the European Union first suggested that the nanospecific provisions should be less comprehensive than in the proposal from the EP, but in the end they accepted them as proposed by the EP. This resulted in the BPR becoming the first piece of European legislation to implement the recommended definition of NMs from the European Commission [15].

The recommended definition of NM by the European Commission states that a NM is: “A natural, incidental or manufactured material containing particles, in an unbound state or as an aggregate or as an agglomerate and where, for 50% or more of the particles in the number size distribution, one or more external dimensions is in the size range 1 nm–100 nm. In specific cases and where warranted by concerns for the environment, health, safety or competitiveness the number size distribution threshold of 50% may be replaced by a threshold between 1% and 50%” [15].

However, only the first part of the recommended definition is adopted by the BPR and the possible replacement of the 50% threshold by a lower one has been omitted. The incidentally created NPs are omitted from the BPR definition of NMs as well. The definition of aggregates and agglomerates in the BPR correspond to the recommended definitions from the European Commission [4].

Besides being the first piece of legislation to adopt the definition of NMs recommended by the European Commission, the BPR is also the first to specify that an approval of an active substance does not cover a corresponding NM form, except when this is it explicitly mentioned [4]. The reasoning for this provision is that NMs will be used as a biocide because of their different properties compared to the bulk form of the substance. These different properties may also result in different toxicities, and therefore NMs require a separate assessment. This means that in order to obtain an authorization of a biocidal product that contains NMs, a specific risk assessment has to be performed for the NM in question and documentation has to be provided to justify that the test performed are relevant and applicable for NMs [4]. Furthermore, the BPR makes it clear that it is not possible to apply for a simplified authorization, which is due to the current lack of adequate risk assessment methods when it comes to biocidal product that contain NMs [4,14].

The BPR further contains requirements regarding labeling of biocidal products and treated articles. In addition to other information, all biocidal products containing NMs must have a label, stating the NMs contained in the product, including information on any specific related risks of the NMs and the word “nano” in brackets following each reference to the given NM [4]. For articles treated with a biocidal product containing NMs, labeling with information of the names of all NMs contained in the product, followed by the word “nano” in brackets, is required [4]. The specific labeling provisions for these materials were included by the EP in recognition of the users’ rights to be informed via adequate labeling given the lack of knowledge on health and environmental impact of NMs [14].

Once a biocidal product is introduced onto the market, each Member State is obliged to submit a report to the European Commission every five years including details of the use of NMs in biocidal products and the potential risk hereof [4].

## 3. Information Requirements for Nanospecific Test Results and Testing Methods

In order to obtain an approval of an active substance and/or a biocidal product, a dossier must be submitted to the competent authority in a chosen member state in the EU. For the active substance, the dossier must fulfill specific information requirements, outlined in Annex II of the BPR, whereas the biocidal product must fulfill the information requirements set out in Annex III [4].

The data requirements, as specified in Annex II and III, comprise a Core Data Set (CDS) and an Additional Data Set (ADS). The CDS is the basis data that, in principle, must be provided for all active substances. There may, however, be cases where it is not possible to generate all data elements belonging to the CDS. This applies in special cases, where physical or chemical properties render it impossible or unnecessary to provide certain data. The ADS to be provided is determined by the physio-chemical properties of the chemical in question, for which type of products the active substance is used, and the exposure patterns that are related to that use (see paragraph 2 in Annex II and Annex III of [4]). For each endpoint in the CDS, at least one key study (or an accepted data waiving justification) must be submitted and the study has to be reliable and adequate to use for the risk assessment [16]. According to Annex II [5] of the BPR the data submitted to support the approval of an active substance must be obtained according to the methods specified in the Test Methods Regulation [17]. For most CDS, the relevant test methods described in the Test Methods Regulation are equivalent to OECD guidelines (see Table 1). If a test method is considered inadequate or not included in this regulation, it is possible to use other scientifically suitable methods; however, justification for the appropriateness of these alternative methods is required. For testing of NMs it is stated that “when test methods are applied to nanomaterials, an explanation shall be provided of their scientific appropriateness for NMs, and where applicable, of the technical adaptations/adjustments that have been made in order to respond to the specific characteristics of these materials” (see paragraph 5 of Annex II and Annex III of [4]).

## 4. OECD and Ecotoxicological Testing of Nanomaterials

The BPR requires nano-specific tests or argumentation for the appropriateness of existing test methods, and specific risk assessment of NMs in order to authorize active substances and/or biocidal products [4]. As mentioned in the introduction, there are currently no actual OECD test guidelines for ecotoxicity testing of NMs [18]. However, to address the challenges of testing NMs the OECD has established the Working Party on Manufactured NMs (WPNM) in 2006, and its subsequent activities (e.g., OECD’s Sponsorship Programme on the Testing on Manufactured NMs) [19].

In 2009, a preliminary review of the current OECD test guidelines for their applicability to NMs was published by the OECD WPNM. For ecotoxicity testing, 24 test guidelines were reviewed with the aim to assess their adequacy in addressing NMs, and in order to identify the need for development of new test guidelines or a revision of the existing test guidelines [20].

The key findings of the review was that all test guidelines contained insufficient guidance for testing of NMs with regards to:

- Material characterization;
- Exposure preparation and delivery of substance to test systems;
- Monitoring of stability and consistency of NMs during the tests; and
- Measurement and use of dose metrics.

It was, however, found that the basic toxicological principles and the test endpoints were adequate for the testing of NMs. The recommendation was therefore not to launch an extensive modification of all OECD guidelines, but rather to address the specific issues related to testing of NMs in a separate document. This work is currently ongoing and the process of drafting this guidance document on aquatic testing of NMs is described elsewhere [12]. It was also highlighted that the terminology used in the existing guidelines in many cases was not applicable for NMs, and that these terms needed to be revised as well [19]. As the preliminary review revealed, one of the primary shortages of the current test guidelines was guidance on sample preparation. As a result of this, OECD published the Guidance Document on Sample Preparation and Dosimetry in 2010, and this was reviewed and amended in 2012 [18]. The guidance outlines important considerations to bear in mind in order to obtain meaningful and reproducible test results [18]. Regarding preparation of samples of NMs for ecotoxicity studies, the guidance document states that the following issues should be taken into consideration: methods of suspension, quantification of media quality and physical-chemical characterization of the NM. Table 2 gives an overview of the most important points and possible suggestions for the preparations of NM samples in aquatic media according to the OECD guidance document [18].

In January 2013, an expert meeting on ecotoxicology and environmental fate of NMs took place in Berlin as a part of the OECD program on the safety of manufactured NMs. The objective of the meeting was to discuss the applicability of the current OECD test guidelines to NMs and provide specific guidance on environmental fate and ecotoxicity testing of these materials. The Guidance Document on Sample Preparation and Dosimetry [18] and the Preliminary review of the OECD Test Guidelines [20] should subsequently be updated based on the recommendations made at the expert meeting, but this update has yet to take place. The conclusions and recommendations from the OECD expert meeting are available from the OECD website [21] and have been published in the scientific literature [22]. It is clear that current OECD Tech Guidelines for ecotoxicity testing, although principally applicable to NMs, are lacking specific guidance on NM specific testing issues, as also recently highlighted in the scientific literature [12]. Additional NM specific guidance will hopefully become available within the foreseeable future as a result of ongoing activities within the OECD. Until then, appropriate testing of NMs is largely based on what can be learned from the open scientific literature, which is the basis of this present analysis.

## 5. Fulfilling the Requirements of the BPR for Copper Oxide

Up to this point, we have discussed the BPR, its requirements, and the OECD evaluation of the applicability of current test guidelines for nanoparticles. In this section, we provide an illustration of how the requirements of the BPR can be implemented for copper oxide nanoparticles (CuNPs).

While silver is the most commonly used biocidal NM [23,24] other metal nanoparticles (NPs) are also being used in products for a biocidal effect [25,26]. Silver, TiO_2_ and ZnO nanoparticles have been studied and characterized to a greater extent than CuO NPs [23,27]. CuO NPs are used as biocides in a range of different products, such as textiles, plastics and paints [28,29,30] and the use of CuO NPs is increasing [31]. The increasing use of NPs for different industrial and commercial applications will inevitably lead to an increased release to the environment [27,32] and once released, aquatic ecosystems are likely to become the receiving bodies for these NMs [32,33]. The actual occurrences and concentrations of CuO NPs in the aquatic environment are, however, unknown due to lack of sufficiently sensitive analytical quantification methods [30]. Various studies of CuO NPs have reported toxicity of CuO NPs towards aquatic organisms such as algae [34], bacteria [26,35], crustaceans [26,35,36], protozoa [37], plants [30], and fish [38] (See Table 3).

**Figure 1:**
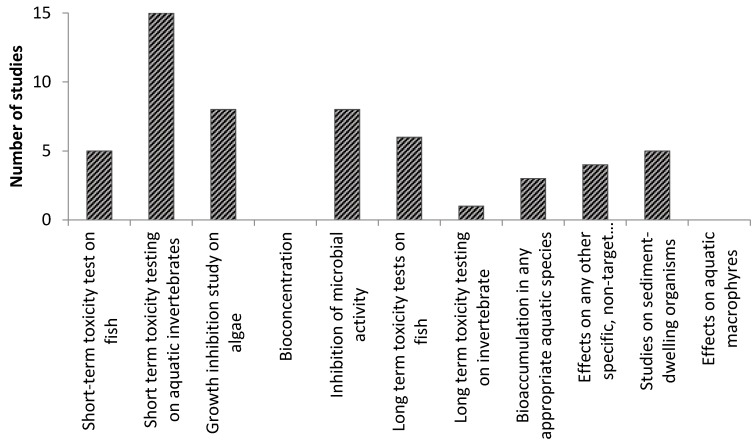
Number of studies potentially fulfilling the Biocidal Product Regulation (BPR) information requirements for ecotoxicity tests.

When it comes to providing data to fulfill the different information requirements in the BPR regarding ecotoxicological effects the number of studies vary to a great extent between different organism groups (see Figure 1). For instance, the data contained in the 15 studies available on “Short-term toxicity testing on aquatic invertebrates” are likely to fulfill the BPR requirement, whereas very few data are available on “bioaccumulation in any appropriate aquatic species”. Eight studies are available on growth inhibition on algae and on inhibition of microbial activity, whereas six are available for short term toxicity towards fish. In regard to assessing the long term toxicity of CuO NP, only one study is available on the long term toxicity on invertebrates, whereas four to six studies are available on the other information requirements except for effects on aquatic macrophytes for which no study is available.

**Figure 2:**
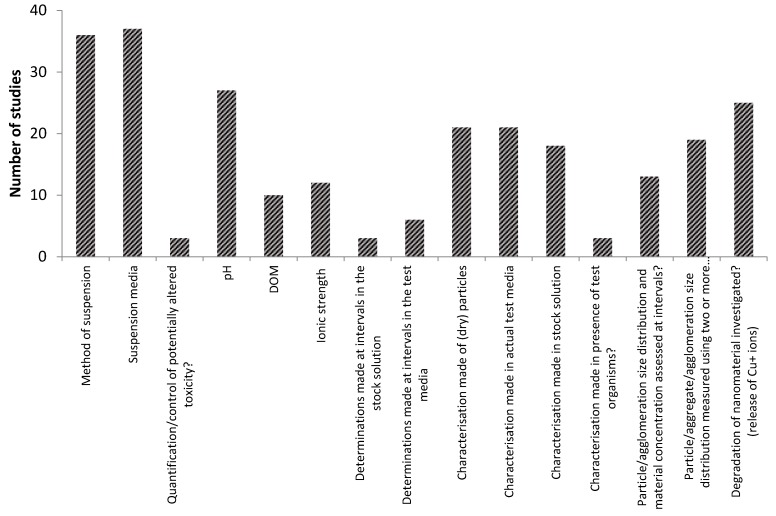
Number of ecotoxicity studies on copper oxide nanoparticles considering the reporting and characterization parameters recommended in the Organisation for Economic Co-operation and Development (OECD) guidance document [18].

When it comes to following the OECD recommendations for characterization and reporting of the 15 different nanospecific parameters shown in Table 3, the studies vary to a great extent. The studies that report most of the parameters, report on seven to nine of these. However about a third of all the studies report on four or less of the parameters suggested by the OECD. The parameters most often reported on are the method of suspension and the suspension media, which is reported in almost three-quarters of all the studies (see Figure 2). pH and the release of copper ions is reported in the a little more than half of the studies. Characterization of (dry) particles, in the actual test media and in the stock suspension is reported in about one third of all the studies. However, only five studies seemingly characterize the tested NPs in their dry form, in the test media as well as in the stock solution. About a third use two methods to characterize their nanoparticles, but very few use more than two methods. The methods most often used are dynamic light scattering, determination of zeta-potential, Transmission Electron Microscopy and/or Scanning Electron Microscopy. Only about 18% report the particle/agglomeration size distribution and materials concentration measured at different intervals during the test.

## 6. Discussion

The BPR is revolutionary as it is the first piece of European legislation to implement the Commission’s recommendation on a definition of nanomaterials and to require a separate dossier to be submitted to European Chemical Agency (ECHA) with all data requirements for the NM used as an active substance or present in biocidal products. However, being first-born is not always a privilege, which certainly seems to be the case for the BPR and nanomaterials.

It is evident from our analysis of studies available in the open literature on the ecotoxicity of CuO NPs, that the BPR requirements can only be partially met with regards to the specific information requirements on ecotoxicological effects. There are only a few published studies that the recommendations put forward in the OECD guidance document [18] into account. This may be due, in part, to lack of reporting on physical-chemical properties, e.g., pH and ionic strength. Other shortcomings might be related technical difficulties in carrying out appropriate and meaningful characterization of NPs. Therefore, nanotechnology-specific biocide test guidelines should be developed.

### 6.1. Challenges for Manufacturers When Testing the Ecotoxicity of Nanomaterials

There are at least four major challenges that manufacturers are facing when attempting to perform nano-specific ecotoxicological testing. The first major challenge relates to materials characterization. Ideally, characterization should be done before, during and after the tests are completed and ideally, the size of the primary NPs and size distribution should be reported along with the crystal structure, surface chemistry, surface charge, solubility and the state of aggregation and agglomeration. It is, however, scientifically and technically very challenging to determine all of these properties especially for NMs that are non-spherical. This is further aggravated due to the tendency of some NPs to aggregate and agglomerate in complex media such as synthetic or natural freshwater media.

Preparation of the NP suspensions by, e.g., sonication and use of solvents before the test is performed have furthermore been observed affect the outcome of the subsequent ecotoxicological testing. Several research projects, for instance Managing Risks of Nanomaterials (MARINA) and the project called “NanoReg” funded by the European Framework Programme 7 [75], have attempted to address this need, however, the most regulatory and/or environmentally relevant preparation method for NP suspensions for ecotoxicity testing still has to be determined. Finally, the composition of the media and the concentration of the NM used in the test as well as dynamic changes during incubation have been reported in the scientific literature to affect the stability of NMs and their aggregation and agglomeration. There is no easy way to deal with the four challenges ecotoxicological testing of NMs but guidance from the OECD on how to address these challenges exist and more is under way. It, however, seems clear that registrants of nanobiocides will have to be explorative in their testing of nanomaterials for some time to come. The development of test guidelines has often taken decades, but hopefully the urgent need, as emphasized by the BPR data requirements, can assist in generating momentum to speed up the process of addressing the scientific and technical challenges of ecotoxicological testing of NMs.

### 6.2. Challenges for Authorities with Regards to Approval of Active Substances under the BPR

There are challenges for the manufacturer or importer of a biocidal NM and there are challenges for the competent authorities responsible for assessing the provided dossier. The BPR mandates nano-specific test requirements and a specific nanobiocide risk assessment be performed by the competent authorities.

These two requirements make implementing the BPR very ambitious and makes the BPR a first mover when it comes to legislation of NMs. At this point in time there is no guidance accompanying the BPR on how to provide nano-specific test results, or how to justify the scientific appropriateness of the current test methods for the testing of NMs [16]. To date, no competent authority has assessed a dossier, based on the nano-specific data requirements according to the available assessment reports [76]; however, one biocidal NM product was approved prior to the establishment of nano-specific testing requirements.

Synthetic Amorphous Silicon Dioxide (SAS) is currently the only NM approved as an active substance under the BPR. The dossier submitted for the approval did not include nano-specific testing for two reasons; firstly, as the dossier for approval was submitted and evaluated before September 2013, the evaluation was based on principles laid out in the Biocidal Product Directive (BPD), which is the directive from 1998 that preceded the BPR, where no nano-specific provisions were included. Secondly, because SAS according to the manufacturers will be present in stable aggregates of 1–6 μm in the active substance, the exposure to primary particles in the nanoscale is not expected during the intended use, and the risk of individual particles does therefore not need to be assessed according to the Assessment Report [77]. Before authorization of an active substance can be granted, an Assessment Report has be prepared by a given Member State and reviewed by the Standing Committee on Biocidal Products, whose opinion will subsequently serve as the basis for European Commission’s decision on whether to approve the substance or not. As this is the first NM to be authorized, the procedure followed in the case of SAS may be precedent for subsequent evaluations of nanobiocides under the BPR. This could create a loophole for not needing to provide hazard data on individual particles, if data showing that the particles form stable aggregates can be provided.

The process for authorization of an active ingredient requires that an Assessment Report be prepared by a given Member State and reviewed by the Standing Committee on Biocidal Products, whose opinion will subsequently serve as the basis for European Commission’s decision on whether to approve the substance or not. Synthetic Amorphous Silicon Dioxide (SAS) is currently the only NM approved as an active substance under the BPR, but was approved prior to September 2013 when nano-specific testing requirements were laid out in the BPR. Secondly, because SAS will be present in stable aggregates of 1–6 μm in the active substance, the exposure to primary particles in the nanoscale is not expected during the intended use, and the risk of individual particles did not need to be assessed according to the Assessment Report [77]. As this is the first NM to be authorized, the procedure followed in the case of SAS may be precedent for subsequent evaluations of nanobiocides under the BPR. That is, if data showing that the particles form stable aggregates can be provided then hazard data on individual particles is not needed. According to Annex II of the BPR [4] justification of the scientific appropriateness of the applied test methods for the testing of the NM should be provided [4]. This was not provided for SAS because it was evaluated according to the BPD prior to the existence of nano-specific requirements in the BPR (1 September 2013). Dossiers submitted after 1 September 2013 should contain justification for all testing.

### 6.3. The Biocidal Product Regulation Will Provide Valuable Data

Although the inclusion of comprehensive nano-specific provisions in the BPR may cause many challenges for both the manufacturers and the regulatory bodies, it will also help to advance the knowledge on the use of nanobiocides on the market and related risks thereof. For instance, the responses from the manufacturers/importers to the Commission inquiry regarding potential nano-biocides, currently supported in the “existing” active substance Review Programme and will provide valuable information on the number and types of nanobiocides that will be in the marketplace in the future. Furthermore, once nanobiocides are approved, member states are required to report information on the use of NMs in biocidal products and their potential risks every five years starting 1 September 2015 [4]. This information will expand the knowledge of the use and potential effects of nano-biocides in the future. The inclusion of the nano-specific provisions in the BPR may also help accelerate the process of developing nano-specific test guidelines and adequate characterization methods.

## 7. Conclusions

Due to the increase in potential use of NMs in biocidal products, nano-specific provisions were implemented in the BPR to assist the development and approval of these products, while ensuring adequate protection of consumers and the environment [14]. However, the lack of nano-specific guidance could conceivably be an obstacle for manufacturers or importers, who wish to introduce new nano-biocides into the market, as official information on establishment of appropriate data is missing. On the other hand, the lack of official guidance may also result in placing of biocidal products containing NMs on the market, where the hazard potential have not been sufficiently investigated, resulting in inadequate protection of humans and/or the environment. Therefore, there is an urgent need to incorporate guidance on nano-specific information requirements into the BPR to fulfill the original purpose of the regulation regarding nano-biocides.

## Figures and Tables

**Table 1:** Information requirements according to Annex II of the Biocidal Product Regulation for ecotoxicity testing, including corresponding test methods as set out in Regulation EC 440/2008 (CDS = Core Data Set, ADS = Additional Data Set, TG = Test Guideline). Data from [4,17].

Test	Specification	Data set	Test method according to regulation EC 440/2008 [17]
**Toxicity to Aquatic Organisms**			
Short-term toxicity testing on fish		CDS *	Test method C1
Short-term toxicity testing on aquatic invertebrates	Test species: Daphnia magna	CDS	Equivalent to OECD TG 202 (2004)
	Other species	ADS	
Growth inhibition study on algae	Effects on growth rate of green algae	CDS	Equivalent to OECD TG 201 (2006) **
	Effects on growth rate of cyanobacteria or diatoms	CDS	Equivalent to OECD TG 201 (2006) **
Bioconcentration	Estimation methods Experimental determination	CDS ***	Equivalent to OECD TG 305 (1996)
Inhibition of microbial activity		CDS	Method C.11.
Further Toxicity Studies on Aquatic Organisms ****			
Long term toxicity tests on Fish	Fish Early Life Stage (FELS) test; Fish short term toxicity test in embryo and sack fry stages Fish juvenile growth test Fish full life circle test	ADS	(b) Equivalent to OECD 212 (1998) (c) Equivalent to OECD TG 215 (2000)
Long term toxicity testing on invertebrate	Daphnia growth and reproductive study Other species reproduction and growth (e.g., Mysid) Other species development and emergence (e.g., Chironomus)	ADS	(a) Equivalent to OECD TG 211 (1998)
Bioaccumulation in any appropriate aquatic species		ADS	
Effects on any other specific, non-target organisms	Non-target organisms: flora and fauna believed to be at risk	ADS	
Studies on sediment-dwelling organisms		ADS	
Effects on aquatic macrophytes		ADS	

Notes: * Data element not required if a valid long-term study on fish is available. ** From EC 761/2009 amending EC 440/2008 [17]. *** Data element not required if it can be demonstrated (on the basis of physical-chemical properties (e.g., logK_ow_ < 3) or other) that the substance has low bioaccumulation potential. **** If the results from the ecotoxicity studies on fate and behavior and/or the intended use(s) of the active substance indicate a risk for the aquatic environment, or if long term exposure is expected, then one or more of the tests mentioned as ADS in the table must be conducted [4].

**Table 2:** Overview of issues and considerations regarding preparation of samples of NMs in exposure media for aquatic ecotoxicity studies. Data from [18].

Issues	Considerations	Recommendations
Method of suspension (e.g., stirring, sonication, grinding, use of solvents and stabilizing agents)	Different suspension methods may significantly alter the NMs *per se* or the toxic properties of the NM.	Best scientific judgment should be used. If there is evidence of altered toxicity, the effects should be controlled or quantified.
Quantification of media quality (e.g., pH, ionic strength and concentration of dissolved organic matter)	Variability in NM properties (e.g., agglomeration/aggregation) depend significantly on media pH, ionic strength and concentration and form of dissolved organic matter.	Media quality determination should be made at intervals sufficient to determine their variability (both in stock suspension and test media); Physical-chemical characterization of NM should be made in the actual test media (whenever possible).
Physical-chemical characterization	Agglomeration/aggregation is likely to occur, which may alter the exposure due to reduced particle counts, surface area or loss of bulk concentration.	Particle size and/or agglomerate size distribution and material concentration must be assessed at intervals during the tests (or at a minimum immediately prior to and after media renewal). Measurements of particle size distribution using two or more methods are desirable. Characterization should be made in the test media in the presence of test organisms (and food if feeding is required).

**Table 3:** Overview of studies published in the open literature on CuO Nanoparticles (NPs) and their relation to the information requirement of the Biocidal Product Regulation (BPR). Furthermore, it is shown which test guidelines the studies have used and which of the nanospecific Organisation for Economic Co-operation and Development (OECD) test and characterization recommendations [18] have been followed.

**Reference**	**[39]**	**[32]**	**[26]**	**[34]**	**[33]**	**[40]**	**[41]**	**[42]**	**[43]**	**[37]**	**[44]**	**[45]**	**[31]**	**[46]**	**[47]**	**[48]**	**[49]**	**[38]**	**[50]**	**[51]**	**[52]**	**[36]**	**[53]**	**[54]**	**[55]**	**[56]**	**[57]**	**[58]**	**[59]**	**[60]**	**[61]**
**Information requirement in accordance with BPR**																															
Short-term toxicity test on fish	x	x				x																							x		
Short term toxicity testing on aquatic invertebrates		x	x					x		x			x		x					x		x	x	x		x					x
Growth inhibition study on algae		x	x	x	x						x						x								x						
Bioconcentration																															
Inhibition of microbial activity					x				x																			x		x	
Long term toxicity tests on Fish							x											x													
Long term toxicity testing on invertebrate																															
Bioaccumulation in any appropriate aquatic species																				x							x				
Effects on any other specific, non-target organisms																x			x		x										
Studies on sediment-dwelling organisms												x		x																	
Effects on aquatic macrophytes																															
**Test Guideline followed**		a		b				c							c	a			a			d	c								d
**OECD ENV/JM/MONO (2012) 40 recommendations**																															
Method of suspension	x	x	x	x	x	x	x	x	x	x		x		x		x	x	x	x	x	x	x		x	x	x	x		x	x	x
Suspension media	x	x	x	x	x	x	x	x	x	x		x	x	x	x	x	x	x	x	x	x	x		x	x	x	x			x	x
Quantification/control of potentially altered toxicity?					x																	x		x							
pH	x	x	x	x	x		x	x		x				x		x		x	x	x	x				x	x	x			x	
Dissolved organic matter	x	x			x			x									x			x											
Ionic strength	x	x			x			x																	x	x	x				
Determinations made at intervals in the stock solution			x														x														x
Determinations made at intervals in the test media			x														x	x		x							x				
Characterization made of (dry) particles	x	x				x		x					x	x	x			x	x	x	x		x						x	x	
Characterization made in actual test media	x	x			x	x	x	x				x		x	x		x		x					x	x					x	
Characterization made in stock solution							x	x	x	x		x					x	x	x			x				x	x		x	x	
Characterization made in presence of test organisms?												x			x									x						x	
Particle/agglomeration size distribution and material concentration assessed at intervals?	x					x	x					x					x	x							x		x		x	x	
Particle/aggregate/agglomeration size distribution measured using two or more methods? [method(s)]	x					x	x	x	x			x		x			x	x	x						x	x	x		x		x
Degradation of nanomaterial investigated? (release of Cu+ ions)	x	x	x	x	x	x	x				x			x	x	x		x	x		x	x	x	x	x					x	x
**Reference**	**[62]**			**[63]**		**[64]**		**[65]**		**[66]**		**[67]**		**[30]**		**[68]**		**[35]**		**[69]**		**[70]**		**[71]**		**[72]**		**[73]**		**[74]**	
**Information requirement in accordance with BPR**																															
Short-term toxicity test on fish	x																														
Short term toxicity testing on aquatic invertebrates																		x		x										x	
Growth inhibition study on algae				x																											
Bioconcentration																															
Inhibition of microbial activity								x				x						x								x					
Long term toxicity tests on Fish	x																					x		x				x			
Long term toxicity testing on invertebrate																		x													
Bioaccumulation in any appropriate aquatic species						x																									
Effects on any other specific, non-target organisms														x																	
Studies on sediment-dwelling organisms						x				x						x															
Effects on aquatic macrophytes																															
**Test Guideline followed**	e													a				f, g, h, i		f								j			
**OECD ENV/JM/MONO (2012) 40 recommendations**																															
Method of suspension	x			x		x				x				x		x				x		x						x		x	
Suspension media	x			x		x								x		x				x		x				x		x		x	
Quantification/control of potentially altered toxicity?																															
pH	x											x		x				x		x		x		x				x		x	
Dissolved organic matter												x				x										x				x	
Ionic strength														x				x		x				x						x	
Determinations made at intervals in the stock solution																															
Determinations made at intervals in the test media																		x													
Characterization made of (dry) particles						x		x										x				x		x		x		x			
Characterization made in actual test media				x		x								x				x						x		x		x			
Characterization made in stock solution				x		x				x						x		x													
Characterization made in presence of test organisms?																															
Particle/agglomeration size distribution and material concentration assessed at intervals?						x																						x		x	
Particle/aggregate/agglomeration size distribution measured using two or more methods? [method(s)]				x				x						x												x					

Notes: (a) ASTM; (b) OECD 201; (c) OECD 202; (d) U.S. EPA; (e) OECD 203/210; (f) ISO6341; (g) ISO 10706; (h) ISO 11348-3; (i) NBR 15411-3; (j) OECD 221.
